# Supplementary material for: Active pharmacovigilance of the seasonal trivalent influenza vaccine produced by Instituto Butantan: A prospective cohort study of five target groups
Source: PLoS One. 2021 Feb 11;16(2):e0246540. doi: 10.1371/journal.pone.0246540 (PMC7877614; doi:10.1371/journal.pone.0246540)
Supplement: S1 File — (DOCX) [file pone.0246540.s001.docx]

**SUPPLEMENTAL INFORMATION 1**

**Active pharmacovigilance of the seasonal trivalent influenza vaccine produced by Instituto Butantan: a prospective cohort study of five target groups**

**Authors:** Tazio Vanni, Beatriz da Costa Thomé, Mayra Martho Moura de Oliveira, Vera Lúcia Gattás, Maria da Graça Salomão, Marcelo Eiji Koike, Maria Beatriz Bastos Lucchesi, Patrícia Emília Braga, Roberta de Oliveira Piorelli, Juliana Yukari Koidara Viscondi, Gabriella Mondini, Anderson da Silva, Heloísa Maximo Espínola, Joane do Prado Santos, Samanta Hosokawa Dias de Nóvoa Rocha, Lily Yin Weckx, Olga Menang, Muriel Soquet, Alexander Roberto Precioso.

**TABLE OF CONTETS**

[Appendix A 3](#_Toc60080435)

[I - Intensity classification 3](#_Toc60080436)

[II - Causal relationship classification 5](#_Toc60080437)

[Table S-0. Frequency of AR among the different target groups (2017 and 2018 studies) recorded during 14 days following immunization. 6](#_Toc60080438)

[Table S-1. Unsolicited adverse reactions in elderly (2017 and 2018) studies recorded during 42 days following immunization. 7](#_Toc60080439)

[Table S-2. Unsolicited adverse reactions in children (2017 and 2018 studies) recorded during 42 days following immunization. 8](#_Toc60080440)

[Table S-3. Unsolicited adverse reactions in pregnant women (2017 and 2018 studies) recorded during 42 days following immunization. 8](#_Toc60080441)

[Table S-4. Unsolicited adverse reactions in postpartum women (2017 and 2018 studies) recorded during 42 days following immunization. 9](#_Toc60080442)

[Table S-5. Unsolicited adverse reactions in healthcare workers (2017 and 2018 studies) recorded during 42 days following immunization. 9](#_Toc60080443)

[Table S-6. All unsolicited adverse reactions (studies 2017-2018) recorded during 42 days following immunization.. 10](#_Toc60080444)

[References 12](#_Toc60080445)

# Appendix A

## I - **Intensity classification**

Based on the guide “Toxicity Grading Scale for Healthy Adult and Adolescent Volunteers Enrolled in Preventive Vaccine Clinical Trials” from the US Food and Drug Administration (USFDA).^1,2,3^

Classification of the intensity of the solicited clinical adverse events.

| **Adverse Event** | **Grade 1** | **Grade 2** | **Grade 3** | **Grade 4** |
| --- | --- | --- | --- | --- |
| Pain at the injection site of the investigational product | Does not interfere  with daily activities | Repeated use of non-narcotic analgesic >24 hours  OR  Mild interference with daily activities | Any use of a narcotic analgesic  OR  Prevent daily activities | Emergency department visit*  OR  Hospitalization |
| Erythema at the injection site of the investigational product ^†^ | 25 – 50 mm | 51 – 100 mm | >100 mm | Necrosis  OR  Exfoliative dermatitis |
| Swelling at the injection site of the investigational product ^†^ | 25 – 50 mm | 51 – 100 mm  OR  Mild interference with daily activities | >100 mm | Necrosis |
| Induration at the injection site of the investigational product ^†^ | 25 – 50 mm | 51 – 100 mm  OR  Mild interference with daily activities | >100 mm | Necrosis |
| Ecchymosis at the injection site of investigational product ^†^ | 25 – 50 mm | 51 – 100 mm  OR  Mild interference with daily activities | >100 mm | Necrosis |
| Pruritus at the injection site of the investigational product | Does not interfere  with daily activities | Mild interference with daily activities | Prevent daily activities | Emergency department visit*  OR  Hospitalization |
| Fever | 37.8 – 38.4°C | 38.5 – 38.9°C | 39.0 – 40.0°C | >40°C |
| Chills | Slight cold sensation; chills, teeth chatter | Moderate chills in the entire body, it requires the use of narcotics | Serious or prolonged, no response to narcotics | ----- |
| Headache | Does not interfere  with daily activities | Repeated use of non-narcotic analgesic >24 hours  OR  Mild interference with daily activities | Any use of a narcotic analgesic  OR  Prevent daily activities | Emergency department visit*  OR  Hospitalization |
| Fatigue | Does not interfere  with daily activities | Mild interference with daily activities | Prevent daily activities | Emergency department visit*  OR  Hospitalization |
| Myalgia | Does not interfere  with daily activities | Mild interference with daily activities | Prevent daily activities | Emergency department visit*  OR  Hospitalization |
| Arthralgia | Does not interfere  with daily activities | Mild interference with daily activities | Prevent daily activities | Emergency department visit*  OR  Hospitalization |
| Pruritus | Mild or localized. One may need to use topical medication | Intense or generalized; intermittent. Changes in the skin due to itching (edema, papules, abrasions, lichenification, crusts). Interferes with daily activities. You may need oral medication | Intense or generalized; intermittent. Prevents self-care, daily activities or sleep. You may need to use systemic corticosteroids or immunosuppressants | Emergency department visit*  OR  Hospitalization |
| Malaise | Does not interfere with daily activities | Interferes little with daily activities | Prevents daily activities | Emergency department visit*  OR  Hospitalization |
| Skin rash (exanthema) ^†^ | Present, but asymptomatic | Symptomatic (pruritus / pain), but interferes little in daily activities | Symptomatic (pruritus / pain), but interferes little in daily activities | Emergency department visit*  OR  Hospitalization |

* It requires 12 hours or more of admission to a ward or emergency department for the management of the adverse event.

^†^ The value recorded should be measured at the largest diameter and as a continuous variable.

^‡^ Specify whether the skin rash is located in any region of the body or if it is widespread.

## **II - Causal relationship classification**

All adverse events had their causal relationship to the investigational product classified according to the adapted classification of the "Uppsala Monitoring Centre" of the World^4^.

Classification of the causal relationship of the adverse events.

| **A reasonable causal relationship** | | | **A causal relationship NOT reasonable** | |
| --- | --- | --- | --- | --- |
| The Adverse Event is considered an Adverse Reaction | | | The Adverse Event cannot be considered an Adverse Reaction | |
| **Certain** | **Probable** | **Possible** | **Unlikely** | **Not related** |
| A clinical event, including a laboratory test abnormality (abnormal value) with a plausible temporal relationship to the administration of the intervention; | A clinical event, including a laboratory test abnormality (abnormal value) with a reasonable temporal relationship to the administration of the intervention; | A clinical event, including a laboratory test abnormality (abnormal value) with a reasonable temporal relationship to the administration of the intervention; | A clinical event, including a laboratory test abnormality (abnormal value) that due to the timing of the administration of the intervention has an unlikely relationship, but not impossible; | A clinical event, including a laboratory test abnormality (abnormal value) that due to the timing of the administration of the intervention has no relationship; |
| It cannot be explained by a concurrent disease or other intervention or medication;  The event must be definitive pharmacologically or phenomenologically (i.e., is an objective and specific disorder or a pharmacologically recognized phenomenon); | It is unlikely to be attributed to a concomitant disease or other medication or intervention; | It can also be explained by a concurrent disease or other medication or interventions; | Another disease or another medication provides a plausible explanation. | Another disease or another medication provides a plausible explanation. |
| The response to an interruption or withdrawal is plausible (pharmacologically, pathologically); | The response to the interruption or withdrawal is clinically reasonable; | There is a lack of information or lack of clarity about the withdrawal or interruption of treatment. |  |  |
| A satisfactory rechallenge procedure if necessary. | A rechallenge procedure is not necessary. |  |  |  |

## Table S-0. Frequency of AR among the different target groups (2017 and 2018 studies) recorded during 14 days following immunization.

|  | **Elderly** | | **ARs** | **Children** | | **ARs** | **Pregnant** | | **ARs** | **Postpartum** | | **ARs** | **HC workers** | | **ARs** | **Participants** | | **Total** |
| --- | --- | --- | --- | --- | --- | --- | --- | --- | --- | --- | --- | --- | --- | --- | --- | --- | --- | --- |
|  | **with AR (n=305)** | | **Total** | **with AR (n=109)** | | **Total** | **with AR (n=108)** | | **Total** | **with AR (n=32)** | | **Total** | **with AR (n=388)** | | **Total** | **with ARs** | | **ARs** |
|  | **n** | **%** | **n** | **n** | **%** | **n** | **n** | **%** | **n** | **n** | **%** | **n** | **n** | **%** | **n** | **n** | **%** | **n** |
| **SOLICITED** |  |  |  |  |  |  |  |  |  |  |  |  |  |  |  |  |  |  |
| **Local** |  |  |  |  |  |  |  |  |  |  |  |  |  |  |  |  |  |  |
| Pain | 87 | 28.5 | 89 | 25 | 22.9 | 25 | 53 | 49.1 | 56 | 10 | 31.3 | 10 | 264 | 68.0 | 270 | 439 | 46.6 | 450 |
| Ecchymosis | 7 | 2.3 | 7 | 3 | 2.8 | 3 | 2 | 1.9 | 2 | 0 | 0.0 | 0 | 2 | 0.5 | 2 | 14 | 1.5 | 14 |
| Erythema | 9 | 3.0 | 9 | 4 | 3.7 | 4 | 3 | 2.8 | 3 | 0 | 0.0 | 0 | 7 | 1.8 | 7 | 23 | 2.4 | 23 |
| Swelling | 13 | 4.3 | 15 | 2 | 1.8 | 2 | 5 | 4.6 | 5 | 0 | 0.0 | 0 | 9 | 2.3 | 9 | 29 | 3.1 | 31 |
| Induration | 10 | 3.3 | 10 | 2 | 1.8 | 2 | 4 | 3.7 | 4 | 2 | 6.3 | 2 | 14 | 3.6 | 14 | 32 | 3.4 | 32 |
| Pruritus | 9 | 3.0 | 9 | 3 | 2.8 | 3 | 7 | 6.5 | 9 | 0 | 0.0 | 0 | 16 | 4.1 | 16 | 35 | 3.7 | 37 |
| **Systemic** |  |  |  |  |  |  |  |  |  |  |  |  |  |  |  |  |  |  |
| Arthralgia | 12 | 3.9 | 12 | 4 | 3.7 | 4 | 19 | 17.6 | 24 | 2 | 6.3 | 2 | 17 | 4.4 | 18 | 54 | 5.7 | 60 |
| Chills | 9 | 3.0 | 9 | 3 | 2.8 | 3 | 9 | 8.3 | 10 | 4 | 12.5 | 5 | 12 | 3.1 | 12 | 37 | 3.9 | 39 |
| Headache | 34 | 11.1 | 38 | 20 | 18.3 | 26 | 50 | 46.3 | 84 | 14 | 43.8 | 25 | 94 | 24.2 | 114 | 212 | 22.5 | 287 |
| Persistent crying | 0 | 0.0 | 0 | 23 | 21.1 | 35 | 0 | 0.0 | 0 | 0 | 0.0 | 0 | 0 | 0.0 | 0 | 23 | 2.4 | 35 |
| Rash | 0 | 0.0 | 0 | 4 | 3.7 | 4 | 2 | 1.9 | 2 | 0 | 0.0 | 0 | 1 | 0.3 | 1 | 7 | 0.7 | 7 |
| Fatigue | 29 | 9.5 | 34 | 12 | 11.0 | 16 | 39 | 36.1 | 53 | 8 | 25.0 | 10 | 63 | 16.2 | 73 | 151 | 16.0 | 186 |
| Fever | 1 | 0.3 | 1 | 29 | 26.6 | 31 | 5 | 4.6 | 6 | 8 | 25.0 | 9 | 2 | 0.5 | 2 | 45 | 4.8 | 49 |
| Irritability | 0 | 0.0 | 0 | 13 | 11.9 | 19 | 0 | 0.0 | 0 | 0 | 0.0 | 0 | 0 | 0.0 | 0 | 13 | 1.4 | 19 |
| Malaise | 14 | 4.6 | 16 | 20 | 18.3 | 23 | 44 | 40.7 | 53 | 3 | 9.4 | 4 | 23 | 5.9 | 28 | 104 | 11.0 | 124 |
| Myalgia | 26 | 8.5 | 26 | 11 | 10.1 | 12 | 17 | 15.7 | 21 | 2 | 6.3 | 2 | 31 | 8.0 | 35 | 87 | 9.2 | 96 |
| Pruritus | 0 | 0.0 | 0 | 1 | 0.9 | 1 | 0 | 0.0 | 0 | 0 | 0.0 | 0 | 2 | 0.5 | 2 | 3 | 0.3 | 3 |
| **TOTAL** | 135 | 44.3 | 275 | 66 | 60.6 | 213 | 77 | 71.3 | 332 | 21 | 65.6 | 69 | 295 | 76.0 | 603 | 594 | 63.1 | 1492 |

## Table S-1. Unsolicited adverse reactions in elderly (2017 and 2018) studies recorded during 42 days following immunization.

| **Unsolicited Adverse Reaction** | **Elderly with ARs* (n=305)** | | **Episodes of ARs*** | **Grade 1** | | **Grade 2** | | **Grade 3** | | **Grade 4** | |
| --- | --- | --- | --- | --- | --- | --- | --- | --- | --- | --- | --- |
|  | **n** | **%** | **n** | **n** | **%** | **n** | **%** | **n** | **%** | **n** | **%** |
| Abdominal pain | 2 | 0.7 | 2 | 2 | 100 | 0 | 0.0 | 0 | 0.0 | 0 | 0.0 |
| Administration site haematoma | 5 | 1.6 | 5 | 4 | 80.0 | 1 | 20.0 | 0 | 0.0 | 0 | 0.0 |
| Administration site pain | 1 | 0.3 | 1 | 1 | 100 | 0 | 0.0 | 0 | 0.0 | 0 | 0.0 |
| Conjunctival hyperaemia | 1 | 0.3 | 1 | 1 | 100 | 0 | 0.0 | 0 | 0.0 | 0 | 0.0 |
| Diarrhea | 1 | 0.3 | 1 | 1 | 100 | 0 | 0.0 | 0 | 0.0 | 0 | 0.0 |
| Dizziness | 1 | 0.3 | 1 | 1 | 100 | 0 | 0.0 | 0 | 0.0 | 0 | 0.0 |
| Dry throat | 2 | 0.7 | 2 | 2 | 100 | 0 | 0.0 | 0 | 0.0 | 0 | 0.0 |
| Erythema outside administration site | 2 | 0.7 | 2 | 2 | 100 | 0 | 0.0 | 0 | 0.0 | 0 | 0.0 |
| Generalized itching | 4 | 1.3 | 4 | 4 | 100 | 0 | 0.0 | 0 | 0.0 | 0 | 0.0 |
| Hyperhidrosis | 1 | 0.3 | 2 | 1 | 50.0 | 1 | 50.0 | 0 | 0.0 | 0 | 0.0 |
| Insomnia | 1 | 0.3 | 1 | 0 | 0.0 | 1 | 100 | 0 | 0.0 | 0 | 0.0 |
| Musculoskeletal pain | 2 | 0.7 | 2 | 2 | 100 | 0 | 0.0 | 0 | 0.0 | 0 | 0.0 |
| Nasal obstruction | 1 | 0.3 | 1 | 1 | 100 | 0 | 0.0 | 0 | 0.0 | 0 | 0.0 |
| Odynophagia | 1 | 0.3 | 1 | 1 | 100 | 0 | 0.0 | 0 | 0.0 | 0 | 0.0 |
| Oropharyngeal pain | 2 | 0.7 | 2 | 2 | 100 | 0 | 0.0 | 0 | 0.0 | 0 | 0.0 |
| Rhinorrhoea | 1 | 0.3 | 1 | 1 | 100 | 0 | 0.0 | 0 | 0.0 | 0 | 0.0 |
| Sneezing | 1 | 0.3 | 1 | 1 | 100 | 0 | 0.0 | 0 | 0.0 | 0 | 0.0 |
| Somnolence | 1 | 0.3 | 1 | 1 | 100 | 0 | 0.0 | 0 | 0.0 | 0 | 0.0 |
| Tremor | 1 | 0.3 | 1 | 1 | 100 | 0 | 0.0 | 0 | 0.0 | 0 | 0.0 |
| Urticaria | 1 | 0.3 | 1 | 0 | 0.0 | 1 | 100 | 0 | 0.0 | 0 | 0.0 |
| Other | 3 | 1.0 | 3 | 2 | 66.7 | 1 | 33.3 | 0 | 0.0 | 0 | 0.0 |
| **TOTAL** | **34** | **11.1** | **36** | **31** | **86.1** | **5** | **13.9** | **0** | **0.0** | **0** | **0.0** |

*ARs: Adverse Reactions

## Table S-2. Unsolicited adverse reactions in children (2017 and 2018 studies) recorded during 42 days following immunization.

| **Unsolicited Adverse Reaction** | **Children with ARs* (n=109)** | | **Episodes of ARs*** | **Grade 1** | | **Grade 2** | | **Grade 3** | | **Grade 4** | |
| --- | --- | --- | --- | --- | --- | --- | --- | --- | --- | --- | --- |
|  | **n** | **%** | **n** | **n** | **%** | **n** | **%** | **n** | **%** | **n** | **%** |
| Cough | 10 | 9.2 | 10 | 8 | 80.0 | 2 | 20.0 | 0 | 0.0 | 0 | 0.0 |
| Diarrhea | 3 | 2.8 | 3 | 3 | 100 | 0 | 0.0 | 0 | 0.0 | 0 | 0.0 |
| Fever | 1 | 0.9 | 1 | 1 | 100 | 0 | 0.0 | 0 | 0.0 | 0 | 0.0 |
| Generalized itching | 4 | 3.7 | 4 | 4 | 100 | 0 | 0.0 | 0 | 0.0 | 0 | 0.0 |
| Influenza | 3 | 2.8 | 3 | 2 | 66.7 | 1 | 33.3 | 0 | 0.0 | 0 | 0.0 |
| Musculoskeletal pain | 6 | 5.5 | 8 | 5 | 63 | 2 | 25.0 | 0 | 0.0 | 1 | 12.5 |
| Nasal obstruction | 1 | 0.9 | 1 | 1 | 100 | 0 | 0.0 | 0 | 0.0 | 0 | 0.0 |
| Pneumonia | 1 | 0.9 | 1 | 1 | 100 | 0 | 0.0 | 0 | 0.0 | 0 | 0.0 |
| Rhinorrhoea | 6 | 5.5 | 7 | 7 | 100 | 0 | 0.0 | 0 | 0.0 | 0 | 0.0 |
| Sneezing | 1 | 0.9 | 1 | 1 | 100 | 0 | 0.0 | 0 | 0.0 | 0 | 0.0 |
| **TOTAL** | **26** | **23.9** | **39** | **33** | **84.6** | **5** | **12.8** | **0** | **0.0** | **1** | **2.6** |

*ARs: Adverse Reactions

## Table S-3. Unsolicited adverse reactions in pregnant women (2017 and 2018 studies) recorded during 42 days following immunization.

| **Unsolicited Adverse Reaction** | **Pregnant women with ARs* (n=108)** | | **Episodes of ARs*** | **Grade 1** | | **Grade 2** | | **Grade 3** | | **Grade 4** | |
| --- | --- | --- | --- | --- | --- | --- | --- | --- | --- | --- | --- |
|  | **n** | **%** | **n** | **n** | **%** | **n** | **%** | **n** | **%** | **n** | **%** |
| Administration site haematoma | 3 | 2.8 | 3 | 3 | 100 | 0 | 0.0 | 0 | 0.0 | 0 | 0.0 |
| Asthenia | 1 | 0.9 | 1 | 1 | 100 | 0 | 0.0 | 0 | 0.0 | 0 | 0.0 |
| Epistaxis | 1 | 0.9 | 1 | 1 | 100 | 0 | 0.0 | 0 | 0.0 | 0 | 0.0 |
| Generalized itching | 11 | 10.2 | 16 | 14 | 88 | 2 | 12.5 | 0 | 0.0 | 0 | 0.0 |
| Hyperhidrosis | 1 | 0.9 | 1 | 0 | 0 | 1 | 100 | 0 | 0.0 | 0 | 0.0 |
| Musculoskeletal pain | 14 | 13.0 | 17 | 6 | 35.3 | 9 | 53 | 2 | 11.8 | 0 | 0.0 |
| Nausea | 1 | 0.9 | 1 | 1 | 100 | 0 | 0.0 | 0 | 0.0 | 0 | 0.0 |
| Petechiae | 1 | 0.9 | 1 | 0 | 0 | 1 | 100 | 0 | 0.0 | 0 | 0.0 |
| Rhinorrhoea | 1 | 0.9 | 1 | 1 | 100 | 0 | 0.0 | 0 | 0.0 | 0 | 0.0 |
| Other | 1 | 0.9 | 1 | 1 | 100 | 0 | 0.0 | 0 | 0.0 | 0 | 0.0 |
| **TOTAL** | **25** | **23.1** | **43** | **28** | **65** | **13** | **30.2** | **2** | **4.7** | **0** | **0.0** |

*ARs: Adverse Reactions

## Table S-4. Unsolicited adverse reactions in postpartum women (2017 and 2018 studies) recorded during 42 days following immunization.

| **Unsolicited Adverse Reaction** | **Postpartum women with ARs* (n=32)** | | **Episodes of ARs*** | **Grade 1** | | **Grade 2** | | **Grade 3** | | **Grade 4** | |
| --- | --- | --- | --- | --- | --- | --- | --- | --- | --- | --- | --- |
|  | **n** | **%** | **n** | **n** | **%** | **n** | **%** | **n** | **%** | **n** | **%** |
| Fever | 1 | 3.1 | 1 | 0 | 0.0 | 1 | 100 | 0 | 0.0 | 0 | 0.0 |
| Headache | 1 | 3.1 | 1 | 0 | 0.0 | 1 | 100 | 0 | 0.0 | 0 | 0.0 |
| Musculoskeletal pain | 2 | 6.3 | 2 | 0 | 0.0 | 2 | 100 | 0 | 0.0 | 0 | 0.0 |
| **TOTAL** | **3** | **9.4** | **4** | **0** | **0.0** | **4** | **100** | **0** | **0.0** | **0** | **0.0** |

*ARs: Adverse Reactions

## Table S-5. Unsolicited adverse reactions in healthcare workers (2017 and 2018 studies) recorded during 42 days following immunization.

| **Unsolicited Adverse Reaction** | **Healthcare Workers with ARs* (n=388)** | | **Episodes of ARs*** | **Grade 1** | | **Grade 2** | | **Grade 3** | | **Grade 4** | |
| --- | --- | --- | --- | --- | --- | --- | --- | --- | --- | --- | --- |
|  | **n** | **%** | **n** | **n** | **%** | **n** | **%** | **n** | **%** | **n** | **%** |
| Abdominal pain | 1 | 0.3 | 1 | 0 | 0.0 | 1 | 100 | 0 | 0.0 | 0 | 0.0 |
| Administration site itching | 1 | 0.3 | 1 | 1 | 100 | 0 | 0 | 0 | 0.0 | 0 | 0.0 |
| Administration site paraesthesia | 1 | 0.3 | 1 | 1 | 100 | 0 | 0 | 0 | 0.0 | 0 | 0.0 |
| Cough | 1 | 0.3 | 1 | 1 | 100 | 0 | 0 | 0 | 0.0 | 0 | 0.0 |
| Dizziness | 2 | 0.5 | 2 | 2 | 100 | 0 | 0 | 0 | 0.0 | 0 | 0.0 |
| Earache | 1 | 0.3 | 1 | 1 | 100 | 0 | 0 | 0 | 0.0 | 0 | 0.0 |
| Erythema outside administration site | 1 | 0.3 | 1 | 0 | 0.0 | 1 | 100 | 0 | 0.0 | 0 | 0.0 |
| Generalized itching | 6 | 1.5 | 6 | 3 | 50.0 | 3 | 50 | 0 | 0.0 | 0 | 0.0 |
| Headache | 1 | 0.3 | 1 | 1 | 100 | 0 | 0 | 0 | 0.0 | 0 | 0.0 |
| Musculoskeletal pain | 4 | 1.0 | 4 | 1 | 25.0 | 3 | 75 | 0 | 0.0 | 0 | 0.0 |
| Nasal congestion | 1 | 0.3 | 1 | 1 | 100 | 0 | 0 | 0 | 0.0 | 0 | 0.0 |
| Nausea | 4 | 1.0 | 4 | 4 | 100 | 0 | 0 | 0 | 0.0 | 0 | 0.0 |
| Odynophagia | 1 | 0.3 | 1 | 1 | 100 | 0 | 0 | 0 | 0.0 | 0 | 0.0 |
| Oropharyngeal pain | 4 | 1.0 | 4 | 3 | 75.0 | 1 | 25 | 0 | 0.0 | 0 | 0.0 |
| Sneezing | 1 | 0.3 | 1 | 0 | 0.0 | 1 | 100 | 0 | 0.0 | 0 | 0.0 |
| Somnolence | 2 | 0.5 | 2 | 1 | 50.0 | 0 | 0 | 1 | 50.0 | 0 | 0.0 |
| Upper respiratory tract infection | 6 | 1.5 | 6 | 2 | 33.3 | 4 | 67 | 0 | 0.0 | 0 | 0.0 |
| Other | 1 | 0.3 | 1 | 1 | 100 | 0 | 0 | 0 | 0.0 | 0 | 0.0 |
| **TOTAL** | **36** | **9.3** | **39** | **24** | **61.5** | **14** | **36** | **1** | **2.6** | **0** | **0.0** |

*ARs: Adverse Reactions

## Table S-6. All unsolicited adverse reactions (studies 2017-2018) recorded during 42 days following immunization..

| **Unsolicited Adverse Reaction** | **Participants with ARs* (n=942)** | | **Episodes of ARs*** | **Grade 1** | | **Grade 2** | | **Grade 3** | | **Grade 4** | |
| --- | --- | --- | --- | --- | --- | --- | --- | --- | --- | --- | --- |
|  | **n** | **%** | **n** | **n** | **%** | **n** | **%** | **n** | **%** | **n** | **%** |
| Abdominal pain | 3 | 0.3 | 3 | 2 | 66.7 | 1 | 33.3 | 0 | 0.0 | 0 | 0.0 |
| Administration site haematoma | 8 | 0.8 | 8 | 7 | 87.5 | 1 | 12.5 | 0 | 0.0 | 0 | 0.0 |
| Administration site itching | 1 | 0.1 | 1 | 1 | 100 | 0 | 0.0 | 0 | 0.0 | 0 | 0.0 |
| Administration site pain | 1 | 0.1 | 1 | 1 | 100 | 0 | 0 | 0 | 0.0 | 0 | 0.0 |
| Administration site paraesthesia | 1 | 0.1 | 1 | 1 | 100 | 0 | 0.0 | 0 | 0.0 | 0 | 0.0 |
| Asthenia | 1 | 0.1 | 1 | 1 | 100 | 0 | 0.0 | 0 | 0.0 | 0 | 0.0 |
| Conjunctival hyperaemia | 1 | 0.1 | 1 | 1 | 100 | 0 | 0.0 | 0 | 0.0 | 0 | 0.0 |
| Cough | 11 | 1.2 | 11 | 9 | 81.8 | 2 | 18.2 | 0 | 0.0 | 0 | 0.0 |
| Diarrhea | 4 | 0.4 | 4 | 4 | 100 | 0 | 0.0 | 0 | 0.0 | 0 | 0.0 |
| Dizziness | 3 | 0.3 | 3 | 3 | 100 | 0 | 0.0 | 0 | 0.0 | 0 | 0.0 |
| Dry throat | 2 | 0.2 | 2 | 2 | 100 | 0 | 0.0 | 0 | 0.0 | 0 | 0.0 |
| Earache | 1 | 0.1 | 1 | 1 | 100 | 0 | 0.0 | 0 | 0.0 | 0 | 0.0 |
| Epistaxis | 1 | 0.1 | 1 | 1 | 100 | 0 | 0.0 | 0 | 0.0 | 0 | 0.0 |
| Erythema outside administration site | 3 | 0.3 | 3 | 2 | 66.7 | 1 | 33 | 0 | 0.0 | 0 | 0.0 |
| Fever | 2 | 0.2 | 2 | 1 | 50.0 | 1 | 50.0 | 0 | 0.0 | 0 | 0.0 |
| Generalized itching | 25 | 2.7 | 30 | 25 | 83.3 | 5 | 16.7 | 0 | 0.0 | 0 | 0.0 |
| Headache | 2 | 0.2 | 2 | 1 | 50 | 1 | 50.0 | 0 | 0.0 | 0 | 0.0 |
| Hyperhidrosis | 2 | 0.2 | 3 | 1 | 33 | 2 | 66.7 | 0 | 0.0 | 0 | 0.0 |
| Influenza | 3 | 0.3 | 3 | 2 | 66.7 | 1 | 33.3 | 0 | 0.0 | 0 | 0.0 |
| Insomnia | 1 | 0.1 | 1 | 0 | 0.0 | 1 | 100 | 0 | 0.0 | 0 | 0.0 |
| Musculoskeletal pain | 28 | 3.0 | 33 | 14 | 42 | 16 | 48.5 | 2 | 6.1 | 1 | 3.0 |
| Nasal congestion | 1 | 0.1 | 1 | 1 | 100 | 0 | 0.0 | 0 | 0.0 | 0 | 0.0 |
| Nasal obstruction | 2 | 0.2 | 2 | 2 | 100 | 0 | 0.0 | 0 | 0.0 | 0 | 0.0 |
| Nausea | 5 | 0.5 | 5 | 5 | 100 | 0 | 0.0 | 0 | 0.0 | 0 | 0.0 |
| Odynophagia | 2 | 0.2 | 2 | 2 | 100 | 0 | 0.0 | 0 | 0.0 | 0 | 0.0 |
| Oropharyngeal pain | 6 | 0.6 | 6 | 5 | 83 | 1 | 16.7 | 0 | 0.0 | 0 | 0.0 |
| Petechiae | 1 | 0.1 | 1 | 0 | 0.0 | 1 | 100 | 0 | 0.0 | 0 | 0.0 |
| Pneumonia | 1 | 0.1 | 1 | 1 | 100 | 0 | 0.0 | 0 | 0.0 | 0 | 0.0 |
| Rhinorrhoea | 8 | 0.8 | 9 | 9 | 100 | 0 | 0.0 | 0 | 0.0 | 0 | 0.0 |
| Sneezing | 3 | 0.3 | 3 | 2 | 67 | 1 | 33.3 | 0 | 0.0 | 0 | 0.0 |

(to be continued)

*TABLE S-6. ALL UNSOLICITED ADVERSE REACTIONS (STUDIES 2017-2018)* *RECORDED DURING 42 DAYS FOLLOWING IMMUNIZATION.* (continuation)*.*

| **Unsolicited Adverse Reaction** | **Participants with ARs* (n=942)** | | **Episodes of ARs*** | **Grade 1** | | **Grade 2** | | **Grade 3** | | **Grade 4** | |
| --- | --- | --- | --- | --- | --- | --- | --- | --- | --- | --- | --- |
|  | **n** | **%** | **n** | **n** | **%** | **n** | **%** | **n** | **%** | **n** | **%** |
| Somnolence | 3 | 0.3 | 3 | 2 | 66.7 | 0 | 0.0 | 1 | 33.3 | 0 | 0.0 |
| Tremor | 1 | 0.1 | 1 | 1 | 100 | 0 | 0.0 | 0 | 0.0 | 0 | 0.0 |
| Upper respiratory tract infection | 6 | 0.6 | 6 | 2 | 33.3 | 4 | 66.7 | 0 | 0.0 | 0 | 0.0 |
| Urticaria | 1 | 0.1 | 1 | 0 | 0.0 | 1 | 100 | 0 | 0.0 | 0 | 0.0 |
| Other | 5 | 0.5 | 5 | 4 | 80.0 | 1 | 20.0 | 0 | 0.0 | 0 | 0.0 |
| **TOTAL** | **124** | **13.2** | **161** | **116** | **72.0** | **41** | **25.5** | **3** | **1.9** | **1** | **0.6** |

*ARs: Adverse Reactions

(end)

## **References**

1. CBER/USFDA/USDHHS Guidance for Industry: Toxicity Grading Scale for Healthy Adult and Adolescent Volunteers Enrolled in Preventive Vaccine Clinical Trials [Internet]. Silver Spring: US Food and Drug Administration; 2007 [cited 2011 Set 29].Available at: <http://www.fda.gov/downloads/BiologicsBloodVaccines/GuidanceComplianceRegulatoryInformation/Guidances/Vaccines/ucm091977.pdf>

2. ICH Clinical Safety Data management: Definitions and Standards for Expedited Reporting [Internet]. Genebra: International Conference on Harmonisation of Technical Requirements ror Registration of Pharmaceuticals for Human Use; 1994 [cited 2012 Oct 15]. E2A. Available at: <http://www.ich.org/fileadmin/Public_Web_Site/ICH_Products/Guidelines/Efficacy/E2A/Step4/E2A_Guideline.pdf>

3. NIC/NIH NCI Common Terminology Criteria for Adverse Events (CTCAE) [Internet]. Available at: <http://evs.nci.nih.gov/ftp1/CTCAE/About.html>.

4. UMC/WHO The use of the WHO-UMC system for standardised case causality assessment [Internet]. Uppsala:The Uppsala Monitoring Centre. Available at: <http://www.who-umc.org/Graphics/24734.pdf>.
